# Physical profiling in a Swedish hockey league team: positional differences and predictors of sprint performance

**DOI:** 10.3389/fspor.2026.1920826

**Published:** 2026-08-21

**Authors:** Ieva Seglina, Simon Östh, Sebastian Bergman, Alexander Ovendal, Henrik Petré

**Affiliations:** Department of Physiology, Nutrition and Biomechanics, The Swedish School of Sport and Health Sciences, Stockholm, Sweden

**Keywords:** age-related performance determinants, body fat percentage, dual-energy x-ray absorptiometry, sprint performance, Swedish Hockey Legaue

## Abstract

**Introduction:**

This study evaluates a professional ice hockey team from the Swedish Hockey League (SHL) using comprehensive player profiling to examine positional differences and to identify how individual age, anthropometric, and physical characteristics predict sprint performance.

**Methods:**

Twenty-three players from one SHL team underwent an off-ice test battery, including anthropometric and physical assessments. The anthropometric assessment included body fat percentage using dual-energy X-ray. Physical performance assessments included isometric mid-thigh pull, vertical jump, off-ice sprints over 5, 10, and 20 meters, and aerobic power. A correlation matrix was computed to examine relationships between physical characteristics. Significant correlations with age were included as predictors for regression models with sprint performance.

**Results:**

Age alone explained 35.7%–43.2% of variance, while fat percentage alone explained 25.9%–34.8% of variance in sprint performance. Together, these two predictors explained 42.6%, 49.1%, and 52.6% of the variance in 5 m, 10 m, and 20 m sprint performance, respectively. Comparisons between playing positions showed no statistically significant differences across all measured variables (*p* > 0.05).

**Discussion:**

These cohort-specific findings indicate comparable physical performance and anthropometric profiles across SHL positions, and that age and body fat percentage are independent predictors of short-distance sprint performance.

## 1 Introduction

Anthropometric and physical performance assessments have long been an integral part of elite ice hockey, offering insight into structural and functional adaptations shaped by distinct training methodologies, competitive demands, and cultural approaches to athlete development (1, 2). This includes both off-ice assessments (i.e., body mass, fat percentage, strength, power, aerobic power) and on-ice assessments (i.e., sprint, agility, skating-specific drills) (1). These assessments are employed to quantify physical fitness, monitor competitive readiness, evaluate performance capacity, and inform evidence-based training interventions (1). While on-ice tests provide higher ecological validity than off-ice testing, they are logistically more demanding and introduce variables that are difficult to control. In contrast, off-ice tests enable higher standardization and more consistently repeatable benchmarks, supporting reliable longitudinal comparisons (3).

A substantial body of research has examined anthropometric and physical performance characteristics across competitive levels, playing positions, and national development systems in ice hockey (4–11). These studies show meaningful subgroup differences; for example, forwards often display higher relative aerobic capacity than defensemen (8, 12), athletes at higher competitive levels demonstrate superior power and speed (9, 13), and players from nations with more competitive hockey infrastructures tend to show superior sprinting capabilities compared to those from less developed systems (10). Collectively, this evidence highlights the importance of context-specific evaluation protocols to ensure accurate and meaningful athlete profiling. Despite this, research involving professional elite players remains limited due to restricted access, leaving a gap in league-specific reference data.

Off-ice sprinting is a well-established indicator of a key physiological quality in ice hockey: the ability to produce rapid, high-force acceleration. This capacity underpins many decisive game actions, as players must repeatedly generate substantial horizontal force over very short distances to create separation, win puck races, and initiate offensive or defensive transitions. Off-ice sprint tests capture this fundamental quality by providing a controlled and reliable measure of an athlete's explosive acceleration potential. Importantly, previous research has demonstrated strong associations between off-ice sprint performance and on-ice skating speed (1, 2, 5, 14–16), suggesting that off-ice acceleration ability reflects a meaningful component of sport-specific skating performance. These consistent relationships support the inclusion of off-ice sprinting as a relevant performance indicator in analytical models aimed at evaluating or predicting ice hockey performance.

The Swedish Hockey League (SHL) ranks among the most competitive and physically demanding ice hockey leagues worldwide, consistently developing athletes who perform at the highest international levels. Although normative data have been reported in other professional ice hockey cohorts (1), comparable data within this specific high-performance league context remain scarce. This limits our ability to gain insight into SHL players capacities and to compare them with players from other top-level professional leagues, such as the National Hockey League (NHL). Multi-team datasets may introduce variability related to heterogeneous training practices. Profiling within a single team may offer a more controlled setting with consistent training and competitive conditions, allowing for a clearer interpretation of strength characteristics and the establishment of internally coherent reference values.

The present study aims to provide context-specific physical profiling of age, anthropometric and physical characteristics in professional elite male hockey players from an SHL team, explore positional differences, and examine relationships between age, anthropometric (body mass, and fat percentage), physical strength characteristics [isometric mid-thigh pull [IMTP] and countermovement jump [CMJ]] and sprint performance. We hypothesize that age, strength related characteristics can predict sprint performance due to age-related declines in neuromuscular power (17, 18). We also hypothesized that fat percentage would increase the models predictive power since non-functional mass increase body mass without contributing to increased force generating capacity (19)

## 2 Materials and methods

### 2.1 Design and procedures

This study used a cross-sectional design to examine anthropometric and physical characteristics of Swedish professional elite male ice hockey players at the highest national level. A standardized test battery including six measurements was designed to determine key physiological characteristics, including muscular strength, sprint, jump performance off-ice and aerobic power. All participants completed the same testing protocol, which was divided into three separate sessions conducted on three different days within two weeks. The testing sessions were separated by at least 48 h, and the testing order was scheduled to minimize the risk of carryover fatigue. During the first occasion, anthropometric measurements and measurements of V̇O_2max_ were performed. During the second occasion, isometric mid-thigh pull (IMTP) and jump measurements were performed. During the third and final occasion, sprint measurements off-ice were performed. The order and content of the sessions were identical for all participants to ensure consistency in data collection. Measurements were performed just before the start of the competitive season, in August, to reflect the period when players typically reached their general physical peak (20), ensuring that the data captured corresponded with real-world performance conditions in professional hockey, unaffected by fatigue, conditioning and adaptation. Participants were asked to avoid strenuous activity for at least 48 h before each testing session and maintain their regular daily nutritional intake. Each testing occasion started with a standardized warm-up including 10 min of continuous cycling at 80 rpm with a braking force of 2.0 kg (160 W) on a Monark cycle ergometer (Model 828, Vansbro, Sweden), followed by 4 min of a dynamic stretching routine focusing on the lower body.

### 2.2 Subjects

Twenty-three male ice hockey players (age 27.1 ± 5.4 years, body mass 87.5 ± 5.3 kg) from one team playing in the highest professional ice hockey league in Sweden, the Swedish Hockey League (SHL), volunteered to participate in the study. They were classified as elite ice hockey players according to their playing level and overall high training volume (21), and professional since they were contracted and financially full-time compensated by the SHL club. All participants had to be injury-free at the time of recruitment. The participants received comprehensive information about the study, including its purpose, potential risks, and anticipated benefits. They completed a health declaration and provided their written informed consent before taking part. The study was approved by the Swedish Ethical Review Authority (Dnr 2025-03038-01) and conducted following the principles outlined in the Declaration of Helsinki.

### 2.3 Procedure

#### 2.3.1 Testing

##### 2.3.1.1 Anthropometrics

*Body mass* was measured using a digital scale (Kern MPI 200K-1, Kern & Sohn GmbH, Germany) with a maximum capacity of 200 kg and a precision of 0.1 kg. All measurements were conducted without shoes and heavy clothes.

*Body fat* percentage was assessed using dual-energy x-ray absorptiometry (DXA) with a Hologic Horizon A scanner (S/N 305579M) and its corresponding software (APEX version 13.6.1.3:3). Participants were instructed to wear athletic shorts and to remove all metal objects (e.g., necklaces, rings, watches) before the scan and were positioned in the center of the scanning bed in an anatomical supine position. The technician ensured proper alignment using the bed's longitudinal midline and secured the ankles with a Velcro strap, resulting in a slight inward rotation of the feet. Subjects were asked to remain still throughout the procedure and to maintain the initial spacing between limbs to prevent overlapping during analysis. DXA is considered a reliable and valid method for assessing total body fat mass and total lean mass (CV = 0.77 to 0.78% and CV = 0.40 to 0.52%) (22).

#### 2.3.2 Physical tests

##### 2.3.2.1 Maximal strength

Lower body maximal strength was determined with an isometric mid-thigh pull test (IMTP). IMTP testing was conducted using dual force plates (ForceDecks FD4000, Vald Performance, Brisbane, Australia). Participants stood upright while gripping a barbell fixed to adjustable chains positioned at mid-thigh level, corresponding to 145° at both the hip and knee joints. Prior to testing, body mass was recorded. Participants then completed three warm-up trials at 50%, 75%, and 90% of perceived maximal effort. Each test trial began with participants applying ∼100 N of pretension, followed by a maximal isometric pull lasting 3 s, initiated by a standardized “3, 2, 1, GO” countdown. Wrist straps were used to secure the hands to the bar, minimizing the influence of grip strength on force output. Body positioning was verified using a goniometer to ensure consistency across trials. A rest period of 3 min was provided between each of the three maximal attempts. Peak force was defined as the highest instantaneous force value recorded during the trial after subtracting the body weight. Force data were sampled at 1,000 Hz and analyzed using Vald ForceDecks software, and the best trial was selected for final analysis.

##### 2.3.2.2 Vertical jump height

Maximal jump height in the Countermovement jump (CMJ) was assessed using the same dual force plate system as the IMTP assessment (ForceDecks FD4000, Vald Performance, Brisbane, Australia), configured with a sampling frequency of 1,000 Hz and a movement detection threshold of 20 N above body weight. After completing two submaximal warm-up jumps with 60 s rest intervals, participants performed maximal CMJ trials with hands placed on their hips (akimbo position) to eliminate arm swing influence.

Standardized instructions were provided to ensure consistency across trials, including: (1) self-selected countermovement depth, (2) maintaining a stationary position for 3 s before jump initiation, (3) executing the movement fluidly without pausing, (4) jumping for maximal height, and (5) landing centrally on the force plates without lateral displacement. Data from the best trial was used for analysis. Variables extracted included jump height (calculated via the impulse-momentum method).

##### 2.3.2.3 Sprint time

Maximal sprint performance off-ice was assessed using a motorized resistance device (1080 Sprint, 1080 Motion AB, Stockholm, Sweden), which recorded time and displacement at a sampling frequency of 333 Hz (24). Participants were connected to the device via a belt fastened around the waist, with the cord from the machine attached to the belt. To minimize slack in the cable, a resistance load of 1 kg was applied in isotonic mode. The auto-start function was enabled, initiating data collection once the athlete reached a velocity threshold of 0.2 m·s⁻¹. The 1080 Sprint system has demonstrated high reliability in measuring sprint performance, with a standard error of the mean (SEM) of 0.04 s (23–25). Sprint trials were conducted over a 20-meter distance, with split times extracted at 5 and 10 m. Each participant performed three maximal sprints from a standing upright start, and the fastest 20-meter trial was used in the final analysis. All participants completed the test on an outdoor artificial-grass soccer field, wearing similar soccer footwear and under comparable environmental conditions with respect to temperature and humidity.

##### 2.3.2.4 Aerobic power

Maximal oxygen consumption (V̇O_2max_) was assessed using a graded exercise test (GET) performed on a motorized treadmill (RL2500 × 100, Rodby, Södertälje, Sweden) to the point of volitional exhaustion. Oxygen uptake was measured continuously using a stationary, automated metabolic cart (Oxycon Pro with mixing chamber, Vyaire Medical GmbH, Hoechberg, Germany), a system known for its high validity and reliability (26). The test protocol began at an individually selected running speed, corresponding to a self-perceived exertion level of 12 on the Borg CR10–20 scale, with the treadmill set at a 1° incline. From this initial workload, speed was increased incrementally each minute until reaching 16–17 km/h. Beyond this point, speed was held constant while the incline increased by 1° per minute until the participant reached volitional exhaustion. Heart rate was continuously monitored throughout the test using a chest-worn pulse sensor (Polar H10, Polar Electro Oy, Kempele, Finland), wirelessly connected to Polar Team software for real-time data acquisition and analysis. The following criteria were used to confirm attainment of V̇O_2max_: heart rate within ±5 beats of age-predicted maximum, rating of perceived exertion (RPE) > 17, respiratory exchange ratio (RER) > 1.1, and a plateau in oxygen uptake despite increased workload. V̇O_2max_ was defined as the highest 60-second average oxygen uptake recorded during the test. A V̇O2 increase below 2.5 mL·kg^−1^·min^−1^ between two 60-second measurements was considered a plateau.

### 2.4 Statistical analyses

Descriptive data were processed in Microsoft Excel (version 2102) and all statistical analyses were performed using R (version 4.3.1; R Foundation for Statistical Computing, Vienna, Austria) in RStudio (version 2023.09.1 + 494). Data was checked for normal distributions using the Shapiro–Wilk test and by visual inspection of the histograms (authors I.S and H.P). Group comparisons between forwards and defensemen were calculated using Welch's two-sided t-test. To determine the magnitude of these differences, Hedges' g effect sizes with 95% confidence intervals were calculated and interpreted as small (0.2), medium (0.5), or large (0.8). A Pearsońs correlation matrix was computed for physical variables across all players. Correlation coefficients was classified as negligible (|*r*| ≤ 0.1), weak (0.1 < |*r*| ≤ 0.4), moderate (0.4 < |*r*| ≤ 0.7), strong (0.7 < |*r*| ≤ 0.9), very strong (0.9 < |*r*| ≤ 1.0) (27).

To examine the predictors of sprint performance, variables showing significant correlations with sprint times were entered as predictors in separate multiple linear regression models for each sprint distance (5 m, 10 m, 20 m). For each model, unstandardized coefficients (*β*) with standard errors, and adjusted *R*^2^ are reported to reflect overall model fit. Semi-partial *r*^2^ was calculated for each predictor using the rsq package in R to quantify the unique variance explained by each variable independent of the other predictors. Variance inflation factors (VIF) were calculated to assess multicollinearity among predictors. Model assumptions were evaluated using the Shapiro–Wilk test for normality of residuals and the Breusch-Pagan test for homoscedasticity. Cook's distance was used to identify the influential cases. The significance level was set at *p* ≤ 0.05. The results are presented as mean ± SD.

## 3 Results

### 3.1 Anthropometric and physical characteristics

The physical characteristics of the players are summarized in Tables 1, 2. Injuries prevented some players from completing all tests (VO_2max_: *n* = 1 injury; IMTP: *n* = 3 injuries and *n* = 1 sickness; jump height: *n* = 1 injury and *n* = 1 sickness; sprint: *n* = 1 injury and *n* = 3 sickness), resulting in slight variations in sample size across variables.

**Table 1:** Anthropometric characteristics of the participants.

Variable	Mean	Position	Mean	*p*-value	Hedges' g (95% CI)
Body Mass [kg] (*n* = 23)	87.4 ± 5.3 (85.1 to 89.8)	Forwards (*n* = 14)	88.4 ± 4.3 (85.5 to 90.5)	0.58	0.24 (−0.59 to 1.06)
		Defensemen (*n* = 9)	86.6 ± 6.8 (81.3 to 91.8)		
Fat [%] (*n* = 23)	15.3 ± 2.9 (14.1 to 16.6)	Forwards (*n* = 14)	15.4 ± 3.3 (13.5 to 17.4)	0.81	0.10 (−0.68 to 0.87)
		Defensemen (*n* = 9)	15.2 ± 2.4 (13.3 to 17)		

Data presented as mean ± standard deviation with 95% confidence intervals.

**Table 2:** Physical characteristics of the participants.

Variable	Mean	Position	Mean	*p*-value	Hedges' g (95% CI)
Jump Height [cm] (*n* = 21)	41.8 ± 4.8 (39.6 to 44)	Forwards (*n* = 14)	41.9 ± 5 (39 to 44.8)	0.855	0.08 (−0.77 to 0.92)
		Defensemen (*n* = 7)	41.5 ± 4.7 (37.1 to 45.9)		
IMTP [N] (*n* = 19)	2,539 ± 376 (2,358 to 2,720)	Forwards (*n* = 13)	2,607 ± 413 (2,358 to 2,856)	0.178	0.60 (−0.27 to 1.46)
		Defensemen (*n* = 6)	2,391 ± 249 (2,129 to 2,653)		
V̇O_2max_ [ml·kg^−1^·min^−1^] (*n* = 22)	57.2 ± 3.7 (55.6 to 58.8)	Forwards (*n* = 14)	56.8 ± 2.8 (55.2 to 58.4)	0.563	−0.26 (−1.12 to 0.61)
		Defensemen (*n* = 8)	57.9 ± 5 (53.8 to 62.1)		
V̇O_2max_ [L·min^−1^] (*n* = 22)	4.99 ± 0.44 (4.79 to 5.19)	Forwards (*n* = 14)	4.98 ± 0.31 (4.80 to 5.17)	0.946	−0.03 (−0.89 to 0.83)
		Defensemen (*n* = 8)	5.00 ± 0.64 (4.47 to 5.54)		
Sprint 5 m [s] (*n* = 19)	1.23 ± 0.07 (1.20 to 1.26)	Forwards (*n* = 12)	1.24 ± 0.07 (1.20 to 1.29)	0.316	0.44 (−0.42 to 1.29)
		Defensemen (*n* = 7)	1.21 ± 0.05 (1.17 to 1.26)		
Sprint 10 m [s] (*n* = 19)	1.99 ± 0.08 (1.95 to 2.02)	Forwards (*n* = 12)	2.00 ± 0.09 (1.94 to 2.05)	0.366	0.40 (−0.46 to 1.26)
		Defensemen (*n* = 7)	1.97 ± 0.06 (1.91 to 2.02)		
Sprint 20 m [s] (*n* = 19)	3.27 ± 0.11 (3.22 to 3.32)	Forwards (*n* = 12)	3.28 ± 0.12 (3.21 to 3.36)	0.516	0.29 (−0.58 to 1.16)
		Defensemen (*n* = 7)	3.25 ± 0.10 (3.15 to 3.34)		

Data presented as mean ± standard deviation with 95% confidence intervals.

Group comparisons between forwards and defensemen revealed no statistically significant differences for any of the variables (*p* > 0.05, Tables 1, 2).

Correlation coefficients for selected age, anthropometric and physical variables are presented in the correlation matrix (Table 3). Age was moderately correlated with 5 m (*p* = 0.013), 10 m (*p* = 0.014) and with 20 m sprint performance (*p* = 0.007). Body mass was moderately correlated with absolute V̇O_2max_ (*p* < 0.001). Body fat percentage showed a moderate negative correlation with 5 m (*p* = 0.050), 10 m (*p* = 0.022), and 20 m sprint time (*p* = 0.026) and with relative V̇O_2max_ (*p* = 0.037). Jump height showed a moderate negative correlation with 20 m sprint time (*p* = 0.026). The strongest correlation was observed between the measures of the same domain: 5 m and 20 m sprint times (strong, *p* < 0.001), 5 m and 10 m sprint times (very strong, *p* < 0.001), and 10 m and 20 m sprint times (very strong, *p* < 0.001), and between absolute V̇O_2max_ and relative V̇O_2max_ (moderate, *p* < 0.002).

**Table 3:** Correlation coefficients between age, anthropometric and physical characteristics of the participants.

Variable	Age [y]	Body Mass [kg]	Fat [%]	Jump Height [cm]	Sprint 5 m [s]	Sprint 10 m [s]	Sprint 20 m [s]	IMTP [N]	V̇O_2max_ [ml·kg^−1^·min^−1^]
Body Mass [kg]	0.27								
Fat [%]	0.05	0.22							
Jump Height [cm]	−0.23	−0.2	−0.38						
Sprint 5 m [s]	0.56*	0.05	0.46*	−0.25					
Sprint 10 m [s]	0.55*	0.16	0.52*	−0.35	0.98*				
Sprint 20 m [s]	0.60*	0.28	0.51*	−0.51*	0.89*	0.96*			
IMTP [N]	−0.007	0.18	0.08	0.21	−0.07	−0.02	−0.05		
V̇O_2max_ [ml·kg^−1^·min^−1^]	−0.13	−0.09	−0.45*	0.05	−0.03	−0.01	0.03	−0.38	
V̇O_2max_ [L·min^−1^]	0.12	0.69*	−0.11	−0.17	0.08	0.18	0.32	0.01	0.62*

* *p* < 0.05.

### 3.2 Linear regression

Multiple linear regression models were conducted for each sprint distance with Age and Fat percentage as predictors (Table 4). All three models were statistically significant (5 m: *p* = 0.005; 10 m: *p* = 0.002; 20 m: *p* = 0.001), explaining between 42.6% and 52.6% of the variance in sprint performance. Regression diagnostics did not indicate violations of model assumptions. Residuals were normally distributed (all *p* > 0.32), variance was homoscedastic (all *p* > 0.37), and VIF values of 1.0 for both predictors across all models indicated no multicollinearity. Given the small sample size, sensitivity analyses were conducted by re-running each model after excluding the case with the highest Cook's distance. Age remained a significant predictor across all models following exclusion, whereas Fat percentage remained significant at 20 m sprint times (*p* = 0.010 to 0.030) but not at 5 m (*p* = 0.031 to 0.272) or 10 m (*p* = 0.010 to 0.104).

**Table 4:** Multiple linear regression models predicting sprint performance (*N* = 19).

Variable	5-m Sprit			10-m Sprit			20-m Sprit		
	*β* (95% CI)	Part *r*^2^	*p*	*β* (95% CI)	Part *r*^2^	*p*	*β* (95% CI)	Part *r*^2^	*p*
Age	0.007 (0.002 to 0.011)	0.357	0.009	0.008 (0.002 to 0.013)	0.378	0.007	0.012 (0.005 to 0.019)	0.432	0.003
Fat%	0.011 (0.001 to 0.021)	0.259	0.031	0.015 (0.004 to 0.026)	0.347	0.010	0.021 (0.006 to 0.037)	0.348	0.010
Adj. *R*^2^	0.426			0.491			0.526		

*β*, unstandardized regression coefficient; 95% CI, 95% confidence interval; Part *r*^2^, semi-partial *r*^2^, representing unique variance explained by each predictor. All predictors were significant at *p* < 0.05.

## 4 Discussion

This is the first study to provide context-specific physical profiling of anthropometric and physical characteristics in professional elite male ice hockey players from an SHL team, explore positional differences, and examine how age, anthropometric, and physical strength characteristics can predict sprint performance. The primary finding was that no differences in anthropometric or physical characteristics between forwards and defensemen were observed, and that age and body fat percentage are key predictors within this cohort of short-distance sprint performance. These findings are partially in line with our hypothesis that age and strength related characteristics predict sprint performance.

SHL players in our sample exhibited *body mass* values comparable to those reported in other professional European leagues, such as the Finnish Liiga and Russian KHL (6, 10), whereas North American players, from the National hockey league and American Hockey League, were generally heavier (∼91.6 vs. ∼87.4 kg) despite showing similar body *fat percentages* (14.7%–15.5% vs. 15.2%) (28). This discrepancy in body mass between European and North American leagues may be attributed to structural and tactical differences between the leagues. The smaller rink dimensions in North America may promote a more physically demanding style of play, characterized by frequent body contact (29), rapid transitions, and tighter spatial constraints. These conditions may favor players with greater mass and muscular robustness, particularly among defensemen (8). In contrast, the larger ice surfaces used in the SHL, and other European leagues may encourage a more fluid and speed-oriented game, where agility and endurance are prioritized over physical size. Thus, the observed differences in body mass may reflect the distinct physiological and strategic demands imposed by each league's playing environment.

*IMTP* performance in the present cohort was comparable to that reported in other strength-dependent sports, including professional rugby (30, 31), underscoring a high level of force-producing capacity relevant to on-ice demands. *CMJ* performance, assessed via force plates, closely matched that of Swiss elite players using the same measurement method (∼42 cm) (32), reflecting a high level of explosive lower-body capability relevant to skating speed and acceleration. Consistent with the literature, our SHL cohort showed relative V̇O_2max_ values of 57.0 [mL·kg^−1^·min^−1^], which falls within the range previously reported for elite ice hockey players in treadmill-based studies [54 to 62 (mL·kg^−1^·min^−1^)] (11). These findings suggest that aerobic fitness for elite players has reached a relatively stable range, suggesting that modern conditioning practices may be approaching the upper range of what can be developed in this capacity within these athletes. Off-ice 5–20 m sprint tests are strong indicators of horizontal power and on-ice speed (1, 5, 14, 15), providing valuable context for evaluating player physical performance. The sprint times recorded in this study, 1.23 s over 5 m, 1.99 s over 10 m, and 3.27 s over 20 m, indicate that players in this Swedish Hockey League team possess a high level of short-distance acceleration and linear sprint capacity, both of which are essential components of modern ice hockey performance (5). Although direct comparisons with external datasets are limited by methodological differences in timing systems, the internal interpretation of these results provides meaningful insight into the players' physical readiness.

The lack of *positional differences* contrasts with earlier longitudinal literature from North American leagues suggesting that defensemen typically possess greater body mass and strength than forwards (8, 12). However, recent tactical developments in elite hockey emphasize speed, transition play, and positional interchangeability, may contribute to homogeneous physical profiles across positions. Hence, the similar anthropometric and physiological characteristics observed may reflect the evolution of positional roles, where modern defensemen are expected to match the speed and mobility of forwards and to participate more in offensive play. However, differences between the leagues may also be influenced by variations in rink size and playing style as mentioned previously.

Age emerged as a consistent predictor of sprint performance across all distances in this sample, explaining 35.7%–43.2% of the variance across models. Moderate correlations (*r* = 0.55–0.60) and significant regression coefficients across 5-, 10-, and 20-m sprint models indicate that older players exhibit slower acceleration. This aligns with evidence that neuromuscular function and explosive force production decline with age, even among elite athletes. Similarly, *body fat percentage* showed moderate correlations with sprint performance across all distances, with higher fat percentage associated with slower sprint times (*r* = 0.46–0.52), uniquely explaining 25.9%–34.8% of the variance. These findings are physiologically plausible, as excess fat mass increases non-functional load and reduces the relative force-to-mass ratio essential for early-phase acceleration, especially at the 20-meter distance. Together, age and fat percentages explained between 42.6% and 52.6% of the variance in sprint performance across the three distances, demonstrating that these variables exert additive and complementary effects on acceleration performance. This combined influence is physiologically coherent: aging reduces neuromuscular efficiency and explosive force production, while excess adiposity increases non-functional mass and lowers the athlete's force-to-mass ratio. Together, these factors diminish the ability to generate high propulsive forces during the early acceleration phase, explaining their consistent contribution across all sprint distances.

*Jump height* showed a significant correlation with 20 m sprint time (*r* = 0.51), but not with 5 m or 10 m sprint times. The absence of a relationship at shorter distances suggests that performance over very short sprints is more strongly influenced by initial acceleration mechanics, starting technique and horizontal force production (33), whereas its moderate correlation with 20 m sprint time may reflect the greater contribution of leg power to maintain the speed over longer distances. *Maximal isometric strength (IMTP)* did not correlate with sprint performance, despite its relevance in other team sports. This may reflect the relatively homogeneous strength levels within this professional cohort, limiting between-player variability. Alternatively, early-phase skating acceleration may depend more on technical efficiency and horizontal force application than on maximal isometric force.

Aerobic power did not show meaningful correlations with any of the measured strength characteristics (5-, 10-, 20-m sprint, IMTP, or CMJ), supporting the notion that these physical characteristics might be largely independent in elite players. *Body fat* percentage showed a moderate negative correlation with relative V̇O_2max_ (*r* = –0.45). Supporting the notion that fat mass acts as non-functional tissue, increasing body mass without contributing to oxygen uptake. Absolute V̇O_2max_, however, was strongly associated with body mass (*r* = 0.69), consistent with fundamental biological principle that larger athletes require greater oxygen consumption due to increased metabolically active tissue.

Despite the practical novelty of the present findings, this study is not without limitations. Although the overall number of participants was relatively high for a study involving professional athletes, the sample size within each subgroup was small. Consequently, these comparisons were potentially underpowered, masking the true positional differences. Therefore, subgroup analyses should be interpreted with caution. Participants were drawn from only one team, which allowed for a controlled comparison within a relatively uniform competitive environment, reducing variability related to different coaching styles and training regimens. Consequently, these data reflect a single club's profile rather than representative values for SHL. While this enhances the internal validity of the study, it also limits the generalizability of the findings to other leagues and playing contexts. Furthermore, the relatively small N limits the statistical power and robustness of the regression models. Sensitivity analyses indicated that the association between Fat percentage and sprint performance at 5 m and 10 m, but not 20 m, was substantially influenced by a single influential case, whereas the effect of age remained robust across all distances. Because this study aimed to analyze one complete SHL team, the sensitivity analysis is presented as a transparency check rather than as a basis for excluding players. Given this instability, the Fat percentage findings at 5 m and 10 m should be interpreted with caution, and replication in a larger sample is needed to determine whether the association with sprint performance holds consistently across distances. A larger, multi-team sample would strengthen the predictive models and provide greater confidence in identifying which physical characteristics are most important for sprint performance in elite hockey. Even so, the approach is well-justified, as establishing preliminary associations within a controlled elite sample provides a critical foundation for subsequent large-scale validation. Finally, the cross-sectional design precludes conclusions about how these characteristics change over the season. Longitudinal monitoring that incorporates both physiological testing and on-ice performance measures would allow future research to examine whether physiological characteristics not only predict off-ice sprint performance but also translate to meaningful differences in on-ice performance.

In conclusion, this study offers a comprehensive profile of age, anthropometric and physical characteristics in professional male ice hockey players from a team competing in the Swedish Hockey League. The findings identify age as a central predictor of short-distance sprint ability within this cohort, explaining a substantial portion of performance variance and underscoring the need for targeted training strategies for older players to maintain sprint performance. Alongside age, body fat percentage was also identified as key factors associated with short-distance sprint performance. Moreover, higher adiposity was significantly related to lower relative V̇O_2max_, suggesting that higher adiposity may negatively influence both anaerobic acceleration capacity and aerobic efficiency. This dual relationship highlights the importance of individualized body composition management within elite hockey programs, where optimizing fat-free mass and minimizing non-functional mass can enhance performance across multiple physiological domains.

### 4.1 Practical applications

The results of this study provide valuable, context-specific insights into age, anthropometric and physical profile of professional ice hockey players from an SHL team. Although the findings from this study should be viewed as preliminary, they provide several useful considerations for applied practice: 1) Training programs may benefit from individualization approaches that extend beyond playing position, as forward and defensemen show comparable anthropometric and physical characteristics; 2) Body-fat management remains important, as higher fat percentage appears to negatively influence sprint performance; and 3) age showed a moderate association with acceleration ability, suggesting that older players may require more targeted work to maintain short-distance speed.

## Data Availability

The raw data supporting the conclusions of this article will be made available by the authors, upon reasonable request.
